# Immunogenic cell death-related risk signature predicts prognosis and characterizes the tumour microenvironment in lower-grade glioma

**DOI:** 10.3389/fimmu.2022.1011757

**Published:** 2022-10-17

**Authors:** Jiayang Cai, Yuanyuan Hu, Zhang Ye, Liguo Ye, Lun Gao, Yixuan Wang, Qian sun, Shiao Tong, Ji’an Yang, Qianxue Chen

**Affiliations:** ^1^ Department of Neurosurgery, Renmin Hospital of Wuhan University, Wuhan, China; ^2^ Central Laboratory, Renmin Hospital of Wuhan University, Wuhan, China; ^3^ Department of Ophthalmology, Tongji Hospital, Tongji Medical College, Huazhong University of Science and Technology, Wuhan, China

**Keywords:** Immunogenic cell death, lower-grade gliomas, prognosis, tumour microenvironment, immunotherapy

## Abstract

Lower-grade glioma (LGG) is a common malignant primary tumour in the central nervous system, and most patients eventually develop highly aggressive gliomas despite comprehensive traditional treatment. Tumour molecular subtypes and prognostic biomarkers play a crucial role in LGG diagnosis and treatment. Therefore, the identification of novel biomarkers in LGG patients is crucial for predicting the prognosis of glioma. Immunogenic cell death (ICD) is defined as regulated cell death that is sufficient to activate the adaptive immune response of immunocompetent hosts. The combination of ICD and immunotherapy might exert a greater and more persistent antitumour effect in gliomas. In our study, we explored the expression, function, and genetic alterations of 34 ICD-related genes. Using 12 ICD-related genes, including IL17RA, IL1R1, EIF2AK3, CD4, PRF1, CXCR3, CD8A, BAX, PDIA3, CASP8, MYD88, and CASP1, we constructed and validated an ICD-related risk signature *via* least absolute shrinkage and selection operator (LASSO) Cox regression analysis. All the information was obtained from public databases, including The Cancer Genome Atlas (TCGA), Genotype-Tissue Expression (GTEx), and the Chinese Glioma Genome Atlas (CGGA) databases. Our results revealed that ICD-high risk groups have a poor prognosis and might be more sensitive to immune checkpoint blockade (ICB) immunotherapy. In addition, ICD-high risk groups were associated with 1p19q noncodeletion, higher WHO grade, wild type IDH, and an immunosuppressive tumour microenvironment. We verified the prognostic value of 12 ICD-related genes in TCGA and CGGA databases. Immunohistochemistry was performed to verify the expression of several ICD-related genes at the protein level. Our study provides a novel and comprehensive perspective to elucidate the underlying mechanisms of LGG prognosis and direction for future individualized cancer immunotherapy.

## Introduction

The most common malignant primary brain tumour in adults is glioma, which accounts for 81% of central nervous system (CNS) malignancies (1). Gliomas are classified into low-grade glioma (LGG) and high-grade glioma (HGG) according to the WHO classification. LGG accounts for 6% of primary tumours of the central nervous system in adults and generally has a better prognosis than HGG. However, comprehensive treatment, including surgical resection combined with chemotherapy and radiation therapy, still cannot avoid treatment resistance and tumour recurrence, and greater than half of LGG patients will eventually develop highly aggressive gliomas (2–4). At present, the WHO classification system of central nervous system tumours increasingly emphasizes the importance of tumour molecular subtypes and prognostic biomarkers for diagnosis and treatment (5). Although IDH mutation, 1p/19q codeletion status, and other prognostic biomarkers have been discovered, these factors are far from sufficient to overcome the dilemma of glioma treatment and prognosis. Therefore, the identification of novel biomarkers in LGG patients is crucial for predicting the prognosis of glioma.

In the latest recommendations of the Nomenclature Committee on Cell Death (NCCA), twelve types of regulatory cell death (RCD) were clearly defined; among them, immunogenic cell death (ICD) was defined as regulated cell death that is sufficient to activate the adaptive immune response of immunocompetent hosts (6). ICD is caused by a relatively limited set of stimuli, including some FDA-approved chemotherapeutic agents, viral infections, specific forms of radiotherapy, and photodynamic therapy. These agents can trigger the timely release of a range of damage-associated molecular patterns (DAMPs) and further stimulate an immune response that is generally associated with the establishment of immune memory (6). In tumours, ICD-promoting therapy can activate tumour-specific immune responses, thereby stimulating the long-term efficacy of antitumour drugs by combining the direct killing of cancer cells and antitumour immunity (7). Notably, two FDA-approved antitumour drugs for use in humans, lurbinectedin (8, 9) and belantamab mafodotin (10), have recently been demonstrated to drive ICD in cancer. In the past few years, various preclinical studies have explored the molecular mechanism of ICD, but few researchers have studied patients in a clinical context to evaluate the possibilities of ICD, such as the identification of biomarkers to classify patients according to their response to ICD immunotherapy, which would be extremely beneficial.

In the present study, we identified ICD-associated biomarkers and developed an ICD-related risk signature that can predict patient prognosis, the immune microenvironment, antitumour drug sensitivity, and the response to immunotherapy in LGG. This risk signature will potentially serve as a significant tool for physicians to make judgements about LGG therapy in the future.

## Materials and methods

### Datasets

Whole-genome RNA-seq expression data and matching clinicopathological data of LGG patients were acquired from The Cancer Genome Atlas (TCGA) database (https://portal.gdc.cancer.gov/) and the Chinese Glioma Genome Atlas (CGGA) database (http://www.cgga.org.cn). A total of 529 LGG samples in TCGA database and 625 LGG (WHO II-III grade) samples in the CGGA database (including CGGA mRNAseq_325 and CGGA mRNAseq_693) were used as training sets and test sets, respectively. In the present study, we eliminated cases with no survival data or those with ≤30 days of survival as these patients potentially die of haemorrhage, intracranial infection, heart failure, or foetal complications rather than LGG. In addition, we used complete mRNA_seq data of 409 normal brain samples from GTEx as a control set, and the “normalizeBetweenArrays” function of the R package “limma” was used to remove multiple batch effects when merging the mRNA_seq data of TCGA, GTEx, CGGA mRNAseq_325 and CGGA mRNAseq_693 (11, 12).

### Identification of differentially expressed genes (DEGs) between low-grade glioma and normal tissues

We combined TCGA-LGG and GTEx databases and analysed DEGs from ICD-related genes using the Wilcoxon test. A P value< 0.05 was established as the criterion. Then, we constructed a coexpression network of these significant genes using GeneMANIA (http://www.genemania.org/), which can identify internal associations in gene sets.

### Consensus clustering

To identify molecular subtypes linked to ICD, we conducted consensus clustering using the R package “ConcensusClusterPlus”. We obtained stable results by assessing the ideal cluster numbers between k = 2–10 1,000 times.

### Identification of differentially expressed genes

The R package “limma” was used to assess differential mRNA expression. We examined adjusted P values to rectify false-positive TCGA data. Adjusted P values less than 0.05 and abs of logFC greater than 0.585 were established as the criteria.

### Functional enrichment analysis of DEGs

To investigate the differential signalling pathways and potential functions of DEGs, we conducted Kyoto Encyclopedia of Genes and Genomes (KEGG) and Gene Ontology (GO) analyses. The R package ‘‘clusterProfiler’’ (13) was used to evaluate KEGG and GO pathways, and q-value or FDR thresholds of<0.05 were considered significant.

### Somatic mutation analysis

We downloaded somatic mutation data of LGG from TCGA database and generated waterfall plots using the R package “Maftools”. Waterfall plots are used to visualize and summarize the mutated gene situation.

### Construction and validation of the ICD-related risk signature

The prognostic value of ICD-related genes was assessed by univariate Cox regression analysis. Then, genes with statistical significance were used to formulate a risk signature through the least absolute shrinkage and selection operator (LASSO) Cox regression analysis. Genes and their regression coefficients were obtained based on the most suitable λ value. The following formula is used to calculate the risk score:


$$\;Risk\;Score\;=\sum_{1}^{n}k_{n}*A_{n}$$

where *A_n_* is the expression level of ICD-related genes, *k_n_* is the regression coefficient of prognosis-related genes, and n is the number of ICD-related genes.

### Prognostic analysis of ICD-related risk signature

The R packages “survminer” and “survival” were utilized to compare the overall survival (OS) between the low and high ICD risk cohorts through Kaplan−Meier (KM) analysis. We also constructed a nomogram that contains relevant clinical parameters and independent prognostic factors using R packages “rms,” “foreign,” and “survival”. Univariate Cox analysis was used to identify prospective prognostic indicators, and multivariate Cox analysis was used to analyse whether the risk score and the nomogram were independent risk factors for OS in LGG. In addition, we generated receiver operating characteristic (ROC) curves of the ICD-related risk signature, nomogram and other clinical risk factors to predict the 1-, 3-, and 5-year overall survival of patients with LGG. Furthermore, we calculated the area under the ROC curve (AUC-ROC), which represents the accuracy of a diagnostic technique. Low accuracy: 0.5< AUC-ROC ≤ 0.7, moderate accuracy: 0.7< AUC-ROC ≤ 0.9, and high accuracy: 0.9< AUC-ROC ≤ 1 (14). Moreover, to evaluate the clinical net benefit of the nomogram, risk, and clinicopathological prognostic factors in predicting 1-, 3-, and 5-year overall survival, we plotted decision curve analysis (DCA) curves by the R package “ggDCA”.

### Clinicopathological relevance of the ICD-related risk signature

LGG patients were separated into high- and low-risk groups, and the differences in gender, age, 1p19q codeletion status, WHO grade and IDH mutation status between the high- and low-risk groups were analysed using the chi-square test. P values< 0.05 were considered significant.

### Tumour-infiltrating immune cells profiles

The CIBERSORT algorithm was used to estimate the abundance profile of 22 immune cell types. Then, we compared the fraction of immune cells between the high- and low-risk groups using the Wilcoxon test. In addition, we calculated the stromal score, immune score, estimate score, and tumour purity of each LGG sample based on the “estimate” package and compared them between the high- and low-risk groups. Furthermore, the expression levels of immune checkpoint (ICP) and human leukocyte antigen (HLA) genes between the low- and high-risk groups were analysed.

### Gene set variation analysis and single-sample gene set enrichment analysis

Gene set variation analysis (GSVA) was performed to study KEGG pathways between the low- and high-risk groups. Single-sample gene set enrichment analysis (ssGSEA) was used to assess immune function scores between the low- and high-risk groups.

### Prediction of drug sensitivity

The Genomics of Drug Sensitivity in Cancer (GDSC) database, Gene Set Cancer Analysis (GSCA) (http://bioinfo.life.hust.edu.cn/GSCA/) and CellMiner (http://discover.nci.nih.gov/cellminer/) were used to analyse the drug sensitivity of ICD-related risk genes. All drugs were approved by the FDA or through clinical trials.

### Human glioma and control brain tissues

All LGG (collected from surgical resection) and control normal brain tissues (collected from patients with traumatic brain injury during emergency surgeries) used in this study were obtained from the Department of Neurosurgery, Renmin Hospital of Wuhan University, China. All patients provided written informed consent, and all specimens had a confirmed pathological diagnosis by pathologists at Renmin Hospital of Wuhan University. The procurement and rational use of specimens in this study were approved by the Institutional Ethics Committee of the Faculty of Medicine, Renmin Hospital Affiliated to Wuhan University (approval number: 2012LKSZ (010) H).

### Immunohistochemistry (IHC)

We immobilized the brain tissues in formalin, cut them into slices and embedded them in paraffin. Sodium citrate (10 mM, pH 6.0) was used for antigen retrieval after deparaffinization and hydration of the tissues. The sections were incubated with 3% H_2_O_2_ for 10 min and blocked with serum for 1 h. Tissues were incubated the primary antibodies (anti-caspase-8 [Proteintech]; anti-MYD88, anti-PERK (EIF2AK3), and anti-CD8 alpha (CD8A) [Servicebio]) (at dilution of 1:1000) at 4°C overnight. Then, samples were incubated with the secondary antibody (at dilution of 1:200) at room temperature for 1 h. Finally, we stained the tissues with DAB (Servicebio) followed by haematoxylin counterstaining. Then, an Olympus BX51 microscope (Olympus) was used to obtain images.

### Western blot analysis

The protein sample was electrophoretically separated on 12% SDS−PAGE gels after measuring the protein concentration and transferred onto 0.45-mm PVDF membranes (Millipore). Then, the membranes were incubated with primary antibodies (anti-caspase-8, anti-MyD88, anti-PERK (EIF2AK3), and anti-CD8A [ABclonal]) at 4 °C overnight followed by HRP-conjugated secondary antibodies (ABclonal). The primary antibody was diluted at 1:1000 and the secondary antibody was diluted at 1:3000. Finally, a ChemiDoc Imaging System (Bio-Rad, USA) was used to capture the images, and ImageJ was used for quantitative analysis. Student’s t test was used for data comparisons between glioma tissues and normal brain tissue groups.

### Statistical analysis

The statistical analysis of bioinformatic sections has been described above in detail. Bioinformatics analyses and R packages were all conducted by R software (version 4.2.0). For the molecular biology experiment, GraphPad Prism 8 was used for the statistical analysis. The means between two groups of normally distributed variables were compared using unpaired Student t-tests. Data that were not normally distributed were compared by the Wilcoxon test. All molecular biology experiment results are reported as means ± SD and were repeated at least three times. *P<0.05, **P<0.01, and ***P<0.001 were regarded as significant.

## Results

### Expression patterns and consensus cluster of ICD-related genes

**Figure 1:**
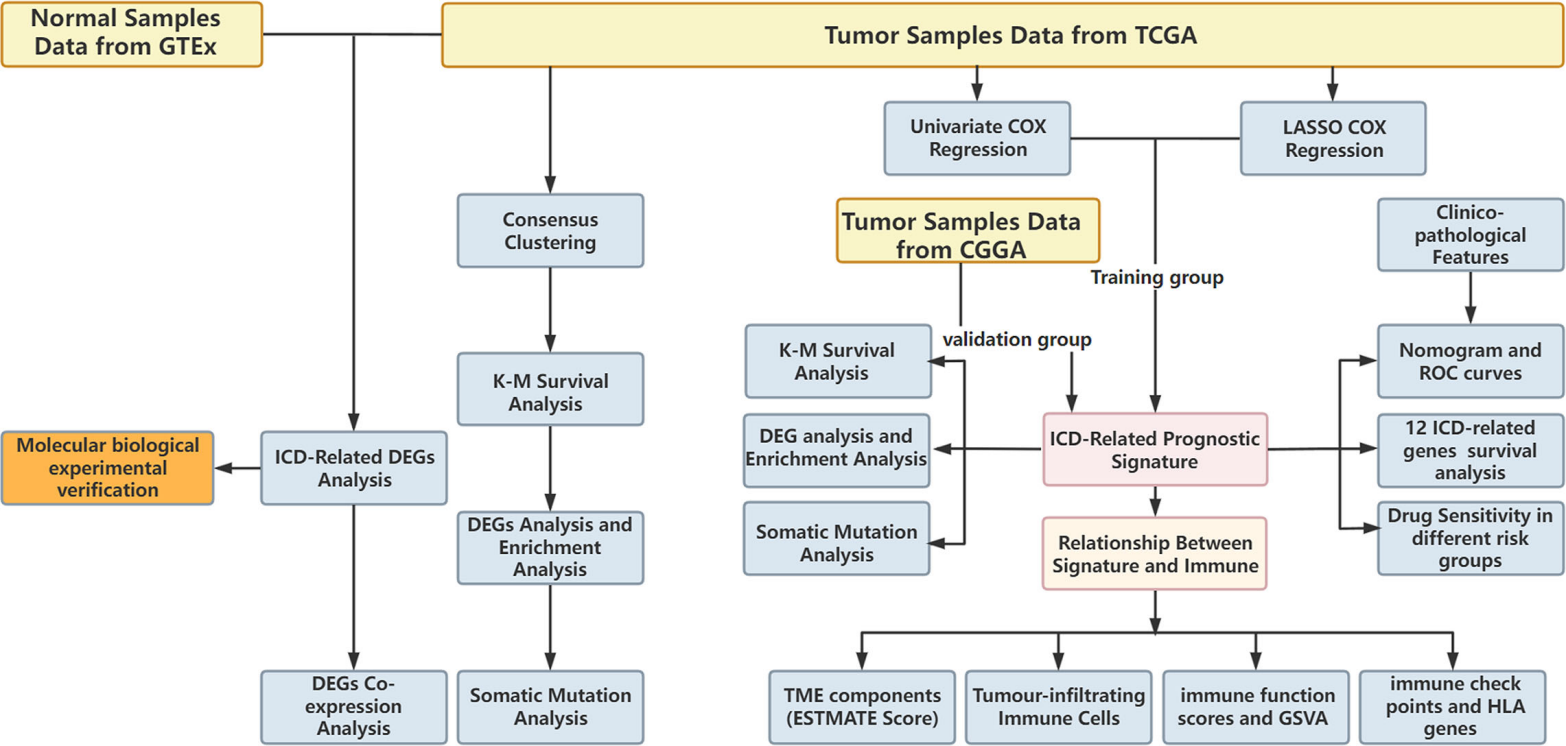
The flow chart of research design.

**Figure 2:**
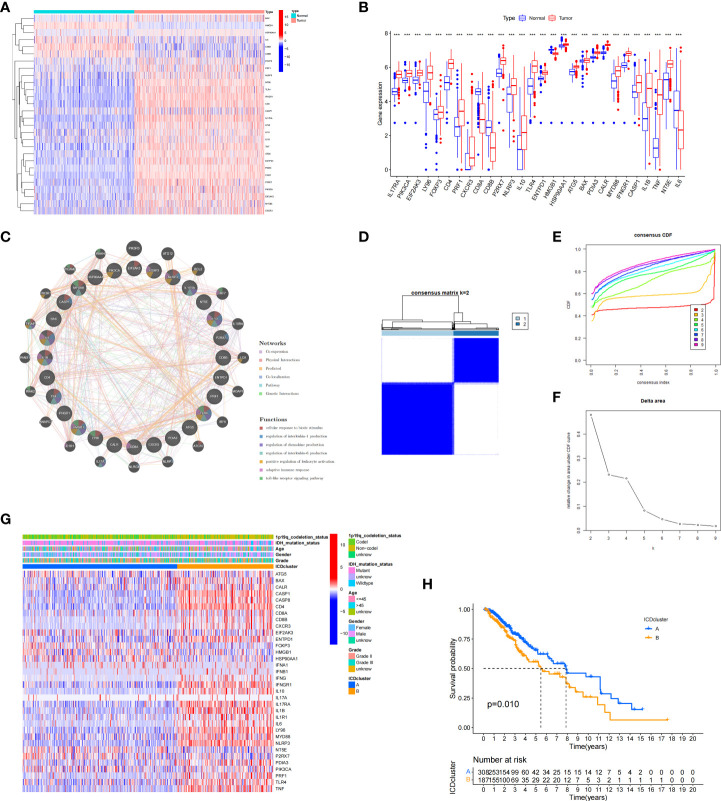
Expression patterns and consensus cluster of ICD-related genes. **(A, B)** Heatmap **(A)** and box plot **(B)** show 28 of 34 ICD genes with significantly different expression profiles between normal and LGG samples in TCGA and GTEx databases, ***P< 0.001. **(C)** Differentially expressed genes and their coexpressed genes were analysed *via* GeneMANIA. **(D)** Heatmap of consensus clustering solution (k = 2) for 34 genes in 529 LGG samples. **(E, F)** The delta area curve of consensus clustering indicates the relative change in the area under the cumulative distribution function (CDF) curve for k = 2 to 10. **(G)** Heatmap of the expression of 34 ICD-related genes in different subtypes. Red represents high expression, and blue represents low expression. **(H)** Kaplan–Meier curves of OS in Cluster A and Cluster B subtypes.

The research flowchart is presented in **Figure 1**. In previous research, Abhishek et al. summarized 34 ICD-related genes through a large-scale meta-analysis (15). We first explored the expression patterns of ICD genes in LGG samples and normal tissues. The results showed that most ICD genes, including IL17RA, PIK3CA, EIF2AK3, LY96, FOXP3, CD4, PRF1, CXCR3, P2RX7, NLRP3, IL10, TLR4, ENTPD1, HSP90AA1, ATG5, BAX, PDIA3, CALR, MYD88, IFNGR1, CASP1, IL1B, TNF, and NT5E, were highly expressed in LGG, whereas CD8A, CD8B, HMGB1, and IL6 were expressed at low levels in LGG (**Figures 2A, B**). Then, we constructed a coexpression network and explored related functions to further reveal the connections among these ICD-related genes (**Figure 2C**). These genes revealed a complex PPI network, which has co-expression of 37.30%, physical interactions of 32.81%, predicted of 19.53%, co-localization of 4.97%, pathway of 4.55%, and genetic interactions of 0.84%. We next identified two clusters (A and B) in TCGA cohort using consensus clustering (**Figures 2D–F**). Difference analysis between these two gene clusters showed that most ICD-related gene expression levels in Cluster B were higher than those in Cluster A (**Figure 2G**). Therefore, we define Cluster A as the ICD-low subtype and Cluster B as the ICD-high subtype. Survival analyses revealed that the ICD-low subtype had a better prognosis (**Figure 2H**).

### Analysis of the differentially expressed genes (DEGs) and functional enrichment analysis in different ICD subtypes

**Figure 3:**
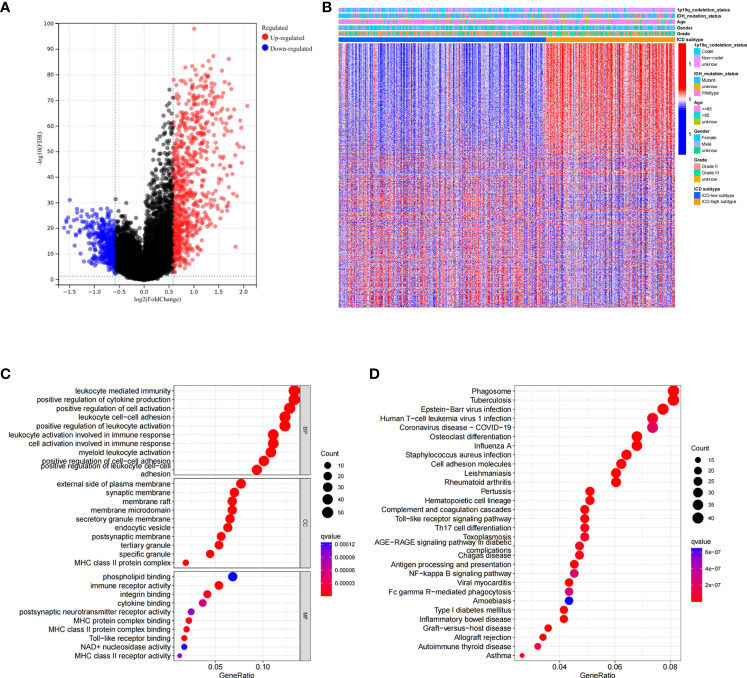
Analysis of differentially expressed genes (DEGs) and functional enrichment analysis in different ICD subtypes. **(A)** Volcano plot of the distribution of DEGs between ICD-high and ICD-low subtypes in TCGA cohort. **(B)** Heatmap of the DEG expression between ICD-high and ICD-low subtypes. **(C, D)** Dots plot of the KEGG **(C)** and GO **(D)** enrichment analysis. The size of the dot represents the gene count, and the colour of the dot represents the q value.

We next conducted difference analysis between the ICD-high subtype and ICD-low subtype (**Figures 3A, B**). A total of 1145 differentially expressed genes were obtained and used for GO and KEGG enrichment analyses. The results showed that these genes are mainly involved in leukocyte-mediated immunity, leukocyte activation involved in immune response, MHC class II protein complex, and immune receptor activity pathways in GO enrichment analysis (**Figure 3C**). KEGG analysis showed that these genes were enriched in phagosome, human T−cell leukaemia virus 1 infection, Toll−like receptor signalling pathway, Th17 cell differentiation, antigen processing and presentation, and other pathways (**Figure 3D**). These results indicated that these ICD-related DEGs played an important role in the immune microenvironment.

### Somatic mutations in ICD-high and ICD-low subtypes

To visualize and summarize mutated genes, we generated waterfall plots for the two gene subtypes. We found different somatic mutation profiles in these subtypes. In ICD-high subtypes, IDH1, TP53, CIC, ATRX, and FUBP1 were the most frequent mutations, responsible for 84.1%, 36.8%, 30.1%, 25.5%, and 12.3% of all mutations, respectively (**Supplementary Figure 1A**). In the ICD-low subtypes, the most frequent mutations included IDH1, TP53, ATRX, TTN, and EGFR, which were responsible for 72.6%, 65.8%, 50.5%, 17.9%, and 10% of total mutations, respectively (**Supplementary Figure 1B**).

### Construction and validation of the ICD risk signature

**Figure 4:**
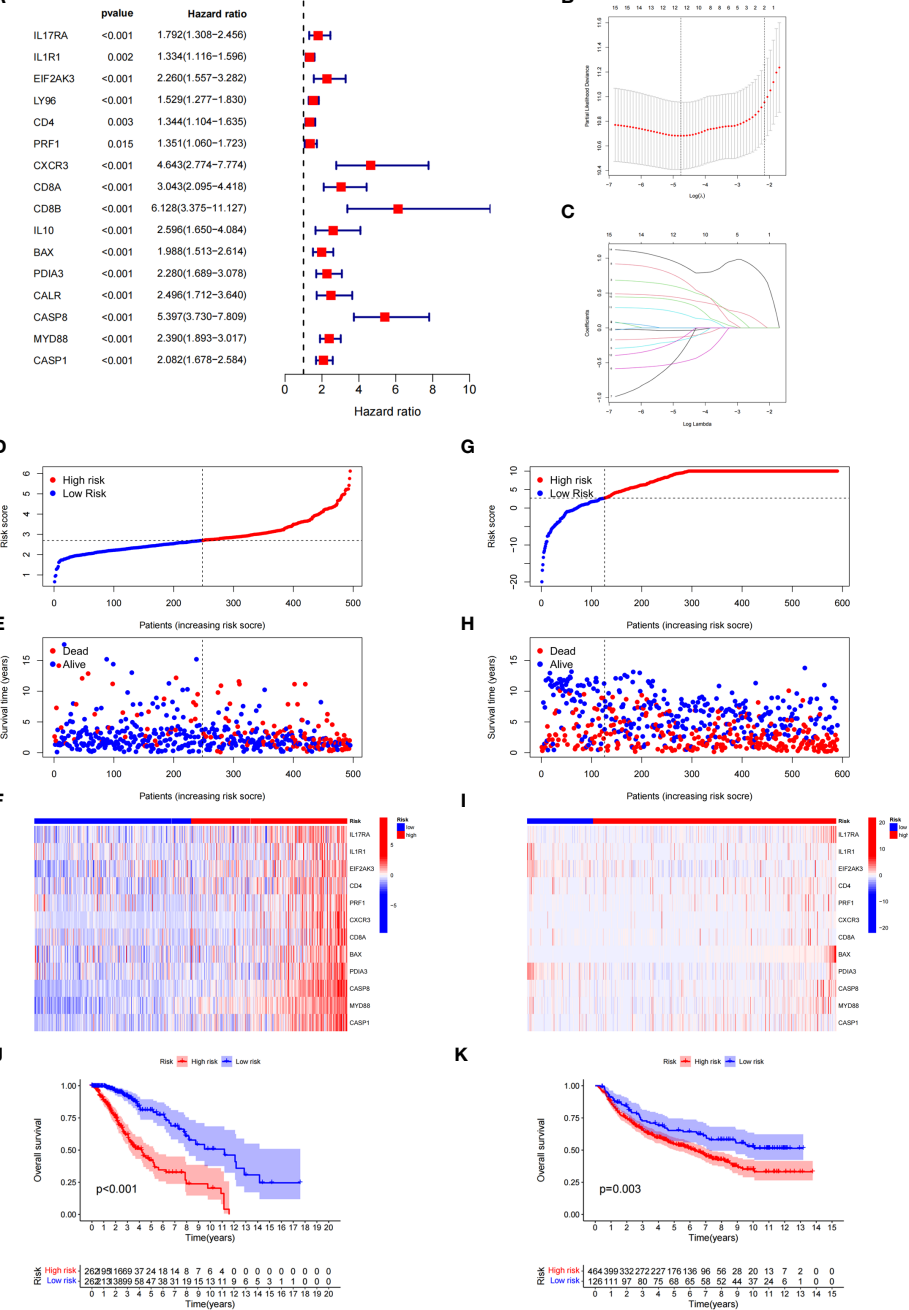
Construction and validation of the ICD-related risk signature. **(A)** Forest plot of overall survival (OS) analysis using univariate Cox analysis for evaluating the prognostic value of the ICD genes. **(B, C)** Lasso Cox analysis identified 16 ICD-related genes most associated with OS in the TCGA dataset. **(D–F)** Risk score distribution **(D)**, survival status of each patient **(E)**, and heatmaps of the prognostic 12-gene signature **(F)** in TCGA database. **(G–I)** Risk score distribution **(G)**, survival status of each patient **(H)**, and heatmaps of the prognostic 12-gene signature **(I)** in the CCGA database. **(J, K)** Kaplan–Meier analysis of the prognostic significance of the risk model in TCGA and CGGA databases.

Based on ICD-related genes, we constructed a prognostic model. Cox univariate analysis found that a total of 16 prognosis-related genes were linked to the OS of patients (**Figure 4A**). Through LASSO regression analysis, 12 ICD-related genes were included in the optimal prediction model (**Figures 4B, C**). **Supplementary Table 1** presents the names of the 12 genes and their corresponding coefficients. In addition, we explored the distributions of risk score, survival status, and risk gene expression in both TCGA (**Figures 4D–F**) and CGGA (**Figures 4G–I**) databases. The results showed that the low-risk cohort included an increased number of alive patients compared with the high-risk cohort in both the TCGA and CGGA databases. Moreover, we conducted KM analysis and found that high-risk patients had a poor prognosis in TCGA database, which was further verified in the CGGA database (**Figures 4J, K**).

### ICD risk score might be an independent factor to predict the overall survival and related to clinicopathological features

**Figure 5:**
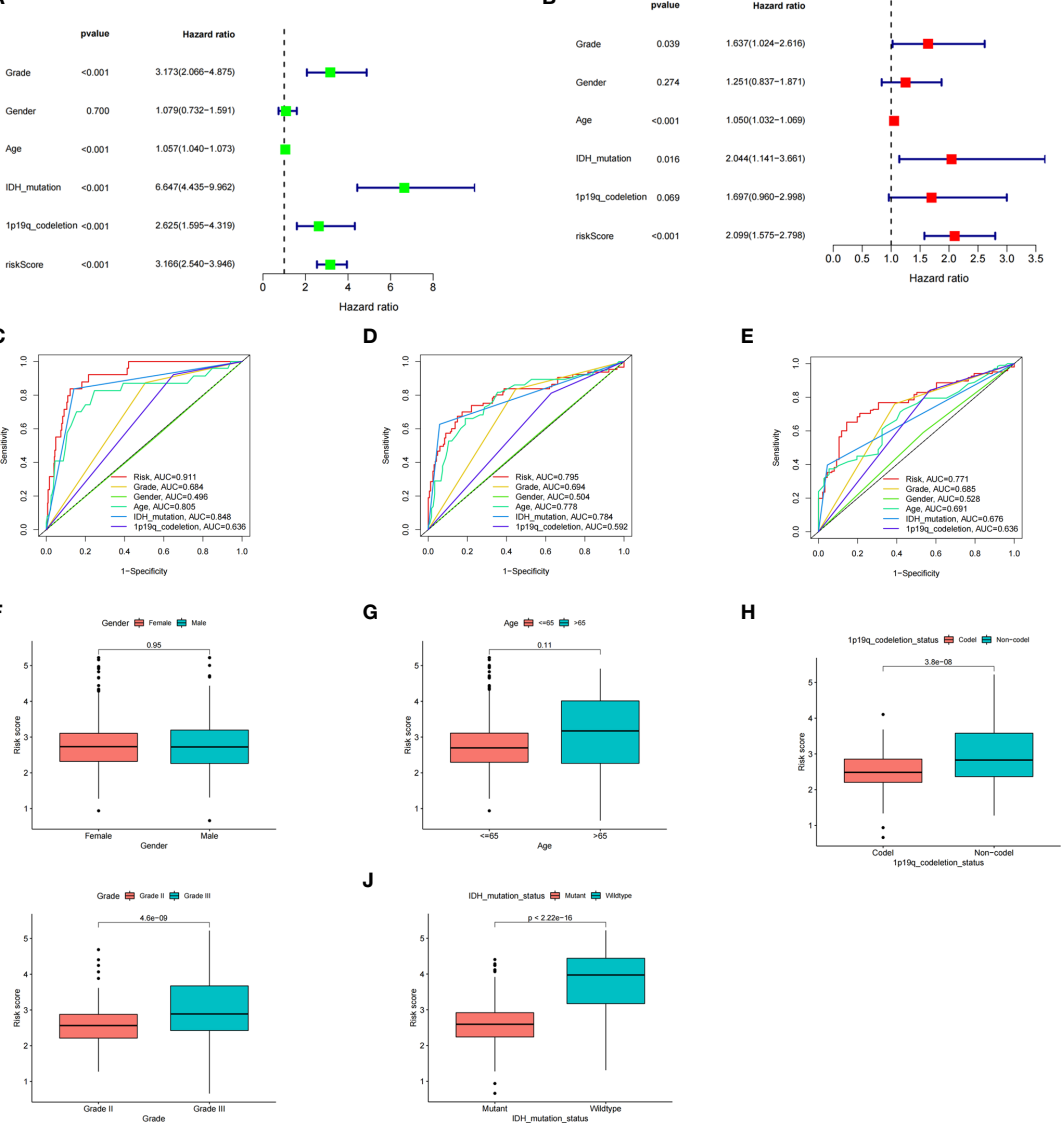
The prognostic value of the risk score and the association between risk score and clinicopathological factors. **(A)** Forest plot of the univariate Cox test evaluating the association of the risk score and clinical factors with patient OS. **(B)** Forest plot of the multivariate Cox analysis identifying independent risk factors for the OS of patients. **(C–E)** The ROC curve of the risk score and clinical factors for predicting 1- **(C)**, 3- **(D)**, and 5-year **(E)** OS. **(F–J)** Distribution of ICD-related risk scores among LGG patients stratified by gender, age, 1p/19q codeletion status, WHO grade, and IDH mutation status in TCGA database.

To better explore the prognostic significance of the ICD risk score, we conducted univariate and multivariate Cox analyses. Our results revealed that the ICD risk score might serve as an independent factor predict the OS of LGG patients (**Figures 5A, B**). Based on the ROC curves of risk scores, WHO grade, gender, age, IDH mutational status, and 1p19q codeletion status, we found that the risk score had a higher area under the ROC curves than other clinical factors in predicting 1-, 3-, or 5-year survival (**Figures 5C–E**). In addition, we explored the relationship between risk scores and other clinical factors and found that 1p19q noncodeletion, higher WHO grade, and wild type IDH status had statistically higher risk scores, and this difference was not noted for age or gender (**Figures 5F–J**).

**Figure 6:**
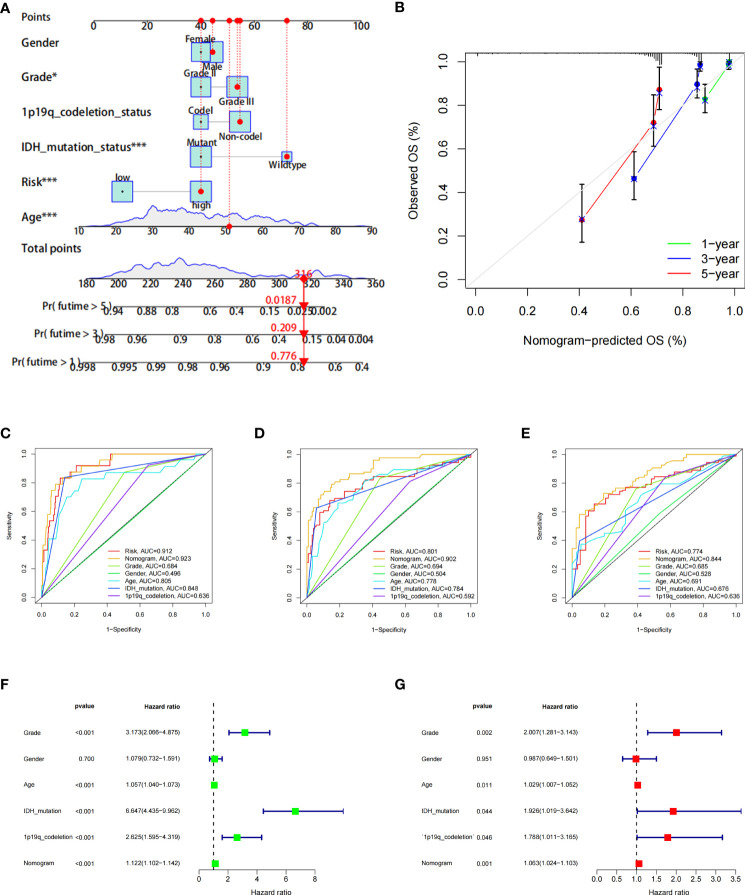
The prognostic value of the risk score combined with clinicopathological features in the OS of patients from the TCGA database. **(A)** Nomogram shows OS in TCGA database of patients. **(B)** The nomogram’s calibration plots. The y-axis represents actual survival, whereas the x-axis represents nomogram-predicted survival. **(C–E)** ROC curve of risk scores and clinicopathological factors for predicting 1- **(C)**, 3- **(D)**, and 5-year **(E)** OS. **(F, G)** Nomogram results based on univariate and multivariate Cox regression analyses.

Furthermore, to more accurately predict the prognosis of LGG patients, we constructed a nomogram based on risk scores and clinical features (**Figures 6A, B**). ROC curves show that nomograms have a better value in predicting the 1-, 3-, or 5-year survival of patients than risk scores alone (**Figures 6C–E**). Univariate and multivariate Cox analyses of the nomogram also showed that this nomogram was an independent factor in predicting the OS of LGG patients (**Figures 6F, G**). Moreover, DCA curves showed that nomograms and risk scores have a better clinical net benefit than other factors in predicting 1-, 3-, or 5-year survival (**Supplementary Figures 2A–C**).

### Analysis of the DEGs, functional enrichment analysis and somatic mutations in the high- and low-risk groups

**Figure 7:**
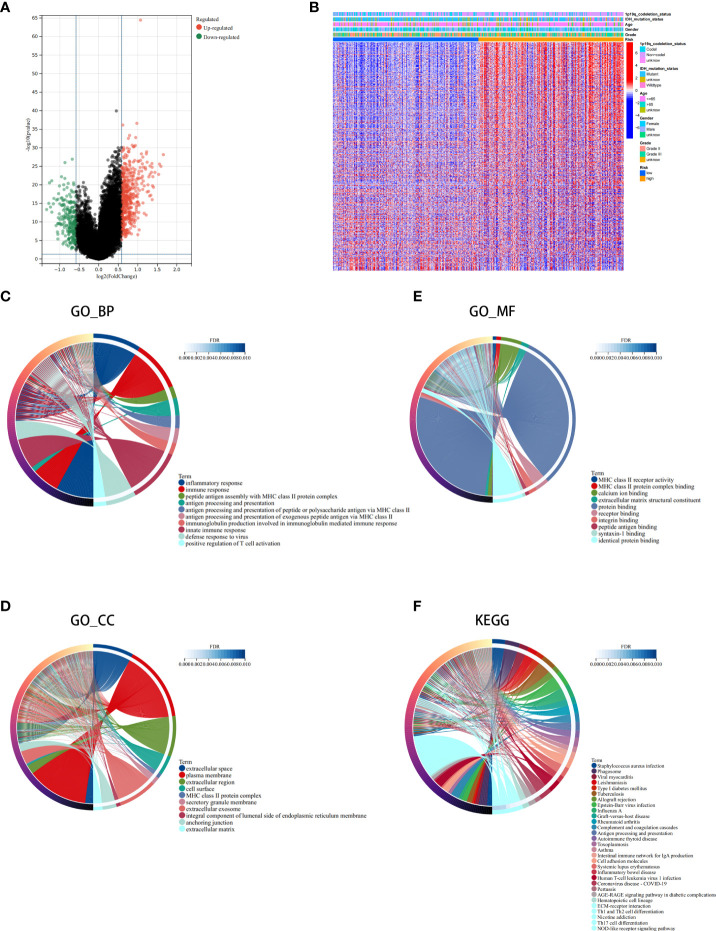
Analysis of the differentially expressed genes (DEGs) and functional enrichment analysis in different ICD risk scores. **(A)** Volcano plot of the distribution of DEGs between the ICD-high and ICD-low risk groups in the TCGA cohort. **(B)** Heatmap of DEG expression between the ICD-high and ICD-low risk groups. **(C–F)** GO **(C–E)** and KEGG **(F)** enrichment analyses. The outermost circle on the right represents the term on the right, and the inner circle on the right represents the FDR of the corresponding pathway.

**Figure 8:**
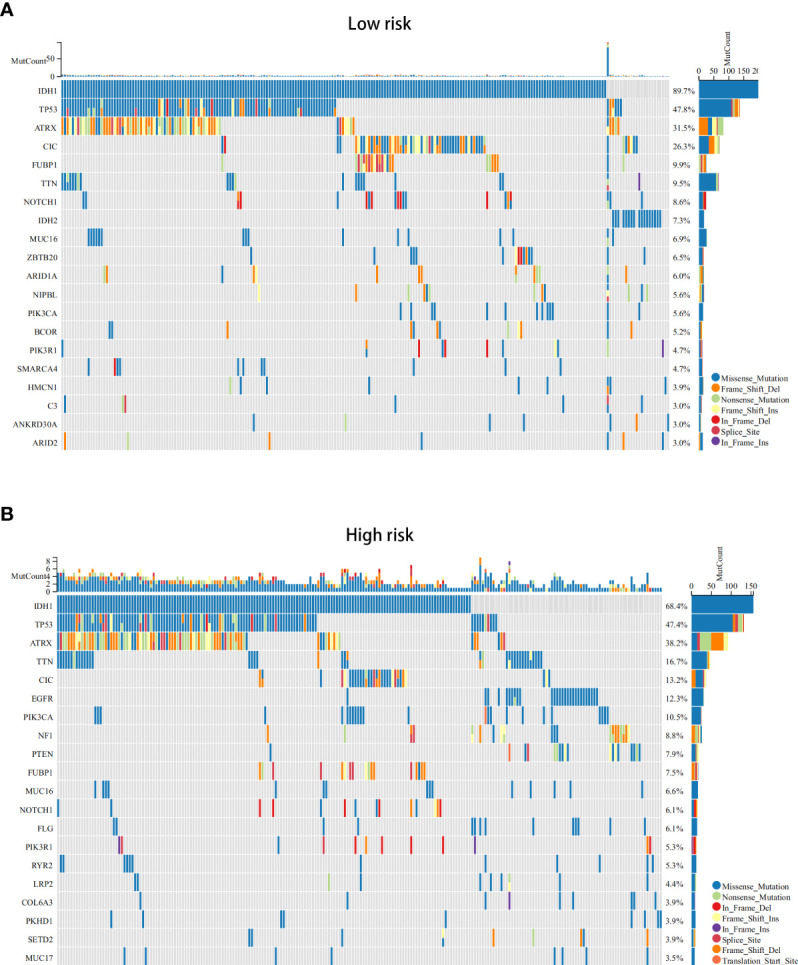
The somatic mutations in the ICD-high and ICD-low risk groups (obtained by LASSO Cox regression analysis). **(A, B)** The top 20 most frequently mutated genes in ICD-high risk groups **(A)** and ICD-low risk groups **(B)** were visualized in waterfall plots.

Next, we conducted differential analysis between the high- and low-risk groups, and a total of 690 DEGs were obtained (**Figures 7A, B**). We conducted GO and KEGG functional enrichment analyses of these genes. KEGG analysis and GO analysis, including biological process (BP), molecular function (MF), and cellular component (CC) analyses, showed that these genes are mainly involved in immune-related pathways (**Figures 7C–F**). In addition, we also explored the mutated genes in the high- and low-risk groups. In the low-risk group, the top 5 mutated genes were IDHI, TP53, ATRX, CIC, and FUBP1. In the high-risk group, the top 5 mutated genes were IDHI, TP53, ATRX, TTN, and CIC (**Figures 8A, B**). Notably, IDH1 mutations occurred more frequently in the low-risk group compared with the high-risk group, which is consistent with the better prognosis of IDH1-mutated LGG patients.

### Tumour microenvironment (TME) landscape in ICD low- and ICD high-risk groups

**Figure 9:**
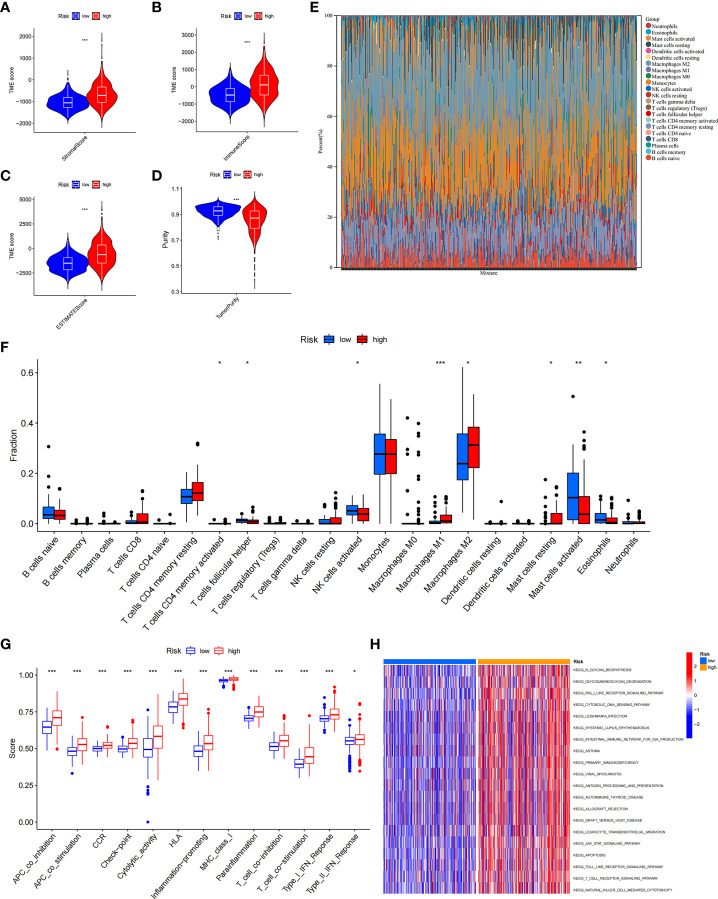
The immune landscape of ICD-high- and ICD-low-risk groups. **(A–D)** Violin plots of the stromal score, immune score, ESTIMATE score, and tumour purity between the ICD-high and ICD-low risk groups. **(E)** Relative percent of immune infiltration in the ICD-high and ICD-low risk groups. **(F)** Box plot visualizes significantly different immune cells between the ICD-high and ICD-low risk groups. *P< 0.05, **P< 0.01, ***P< 0.001. **(G)** Box plots of the difference in known functions related to immune modulation between the ICD-high and ICD-low risk groups. *P< 0.05, ***P< 0.001. **(H)** GSVA enrichment heatmap for ICD-high and ICD-low risk groups.

Growing evidence suggests that tumour-infiltrating immune cells play an essential role in the tumour microenvironment. We investigated stromal score, immune score, estimate score, and tumour purity between the ICD low- and ICD high-risk groups. We found that stromal score, immune score, and ESTIMATE score were higher and tumour purity was lower in high-risk samples (**Figures 9A–D**). Next, we assessed the relative fraction of immune infiltration in 22 types of immune cells using the “CIBERSORT” algorithm, and the results of LGG patients from TCGA dataset were summarized (**Figure 9E**). In detail, the proportions of activated memory CD4 T cells, M1 and M2 macrophages, and resting mast cells were increased in the high-risk group, whereas the proportions of follicular helper T cells, activated NK cells, activated mast cells, and eosinophil cells were lower in the high-risk group (**Figure 9F**). These results indicated that risk scores might be associated with immune infiltration levels to affect LGG patient prognosis.

**Figure 10:**
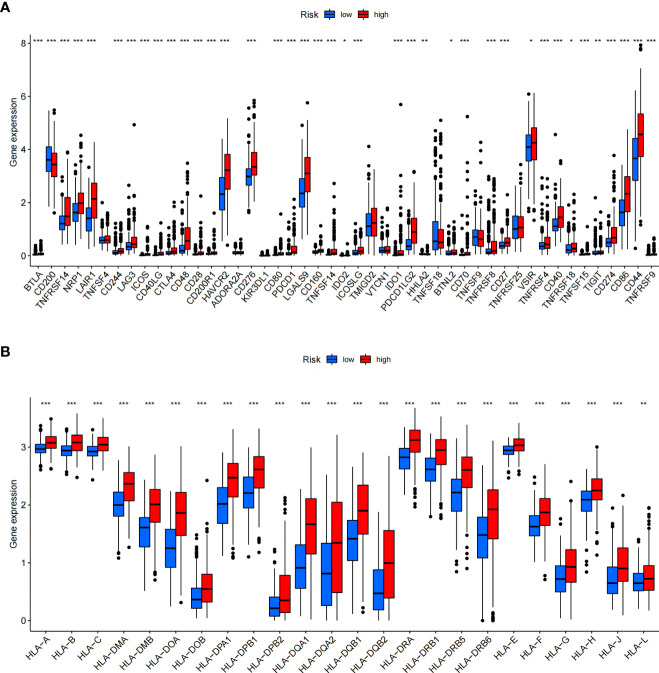
Differential expression of immune checkpoints and HLA genes. **(A, B)** Box plots of differentially expressed immune checkpoints **(A)** and HLA genes **(B)** between the ICD-high and ICD-low risk groups. *P< 0.05, **P< 0.01, ***P< 0.001.

In addition, we assessed the activities and abundances of functions and pathways based on the ssGSEA scores. Our results showed that the high-risk group had higher ssGSEA scores in various immune functions (**Figure 9G**). GSVA revealed that the high-risk group exhibited increased activity in primary immunodeficiency, antigen processing and presentation, T-cell receptor signalling pathway and other immune-related pathways (**Figure 9H**). Furthermore, considering the importance of immune checkpoints (ICPs) and human leukocyte antigen (HLA) genes in anticancer immunity, we analysed the expression of 47 ICPs and 24 HLA genes in different risk groups. The results showed that almost all of the ICP and HLA genes were significantly upregulated in the high-risk group (**Figures 10A, B**). The above results suggest that the risk score value is strongly related to the expression level of ICPs and HLA genes, which may represent potential biomarkers for immunotherapy.

### Prognostic value of ICD-related risk genes and prediction of drug sensitivity

**Figure 11:**
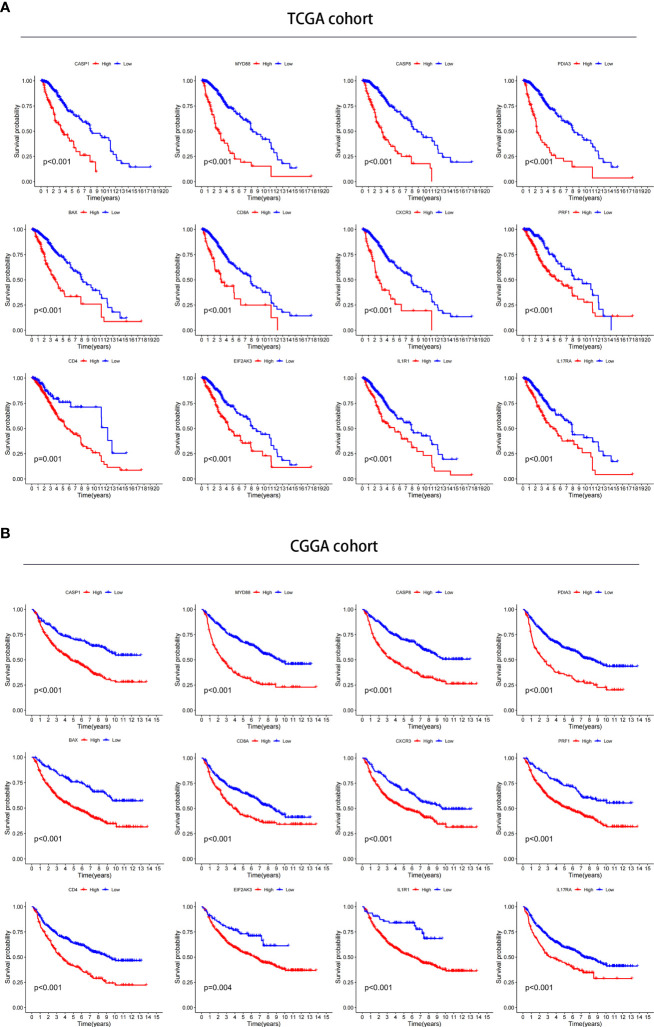
Verification of the prognostic value in TCGA and CGGA databases. **(A, B)** Kaplan–Meier analysis of 12 ICD-related risk genes for patients in TCGA **(A)** and CGGA **(B)** databases.

**Figure 12:**
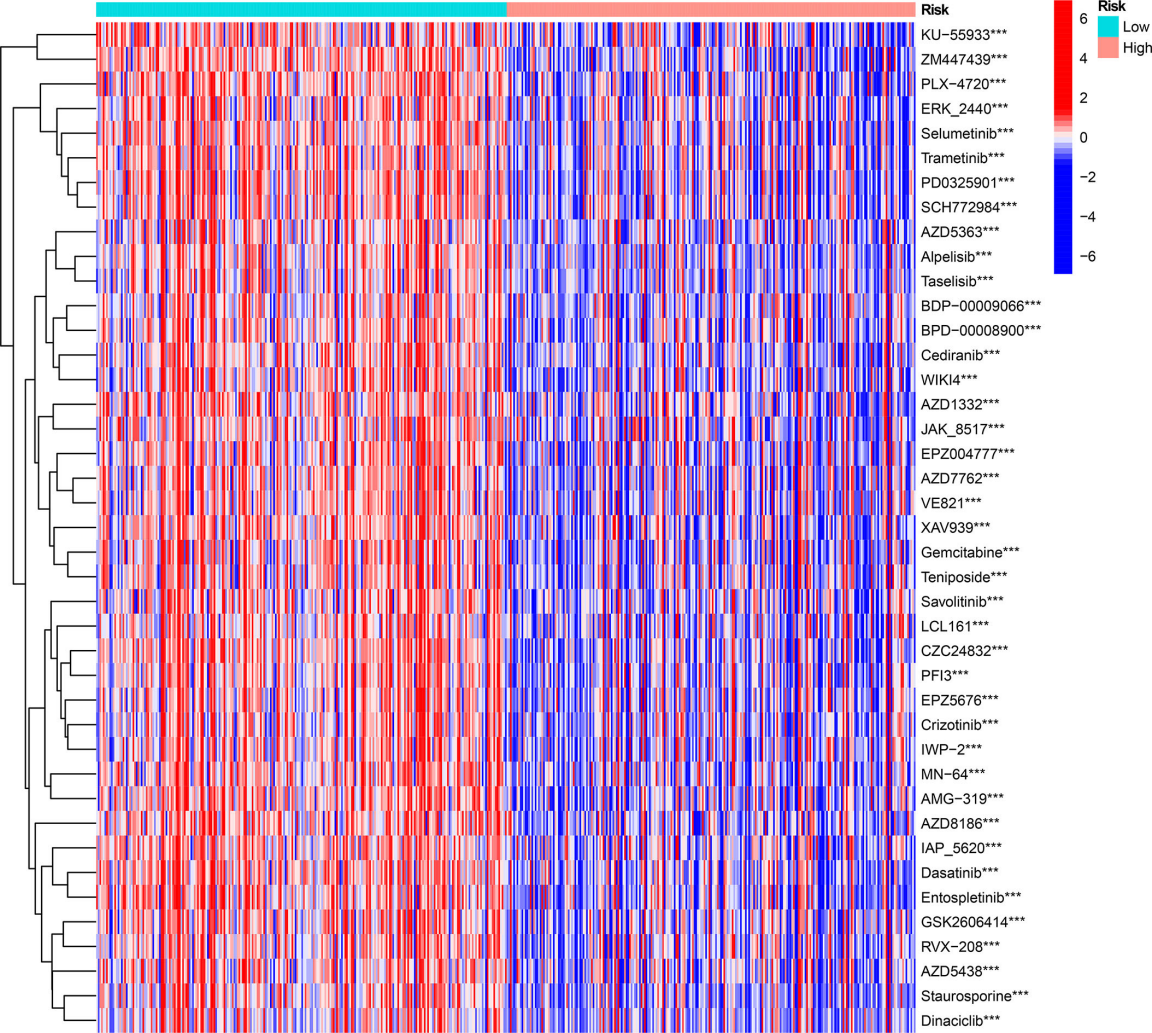
The drug sensitivity prediction and comparison of ICD-high and ICD-low risk groups from the GDSC database are sum up in a heatmap. ***P < 0.001.

We investigated the prognostic value of 12 ICD-related genes involved in the prediction model. Kaplan−Meier survival analysis indicated that LGG patients with high expression of the 12 ICD-related genes had a poor prognosis in both TCGA and CGGA databases (**Figures 11A, B**). Subsequently, we predicted drug sensitivity using the GDSC database and found that high-risk patients were more sensitive to various drugs, and we listed the top 41 drugs in the heatmap (**Figure 12**). Moreover, we also explored the relationship between the expression levels of these 12 ICD-related genes and drug sensitivity in pan-cancer using the CellMiner and GSCA databases (**Supplementary Figure 3** and **Supplementary Figure 4**).

### Validation of ICD-related gene expression levels in clinical tissue

**Figure 13:**
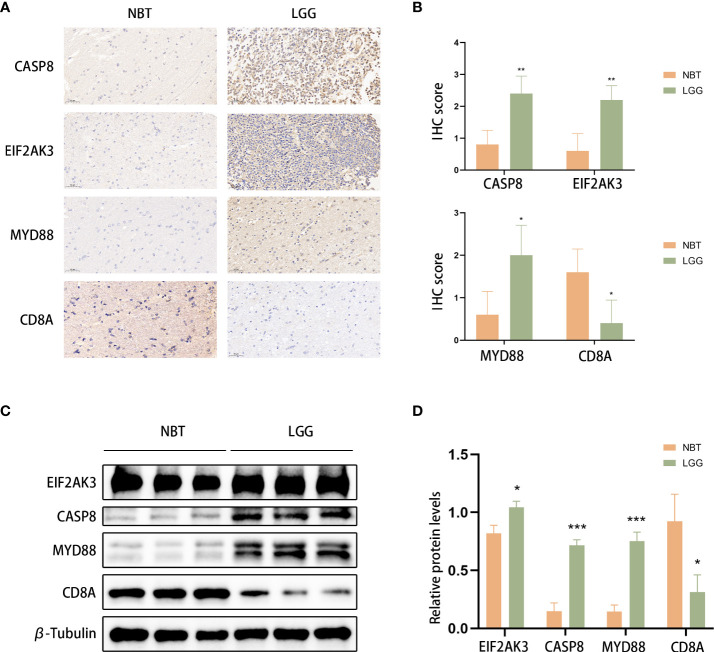
CASP8, MYD88, EIF2AK3, and CD8A expression was verified. **(A, B)** Representative IHC staining images **(A)** and IHC scores **(B)** for CASP8, MYD88, EIF2AK3, and CD8A in clinical tissues. Scale bars: 50 μm. Normal brain tissue: n = 5, LGG: n=5.**(C, D)** Western blotting was used to detect the protein expression levels of EIF2AK3, Caspase 8, CD8A, and MyD88 in LGG (n=3) and normal brain tissues (n=3). *P< 0.05, **P< 0.01, ***P< 0.001.

We used LGG and control normal brain tissues (NBT) to validate the expression levels of ICD-related genes. We selected the 4 genes with the highest coefficients (including CASP8, CD8A, EIF2AK3, and MYD88) in the risk model to perform immunohistochemistry staining. IHC results showed that CASP8, EIF2AK3, and MYD88 had higher expression levels in LGG tissues, whereas CD8A exhibited the opposite trend, which was consistent with the results obtained with TCGA database (**Figures 13A, B**). Similar results were obtained based on Western blot analyses (**Figures 13C, D**).

## Discussion

For a long time, people have been exploring better treatment methods for glioma, especially in LGG, which has a relatively low degree of malignancy and is a relatively promising tumor for cure in glioma, but the traditional surgical resection combined with chemotherapy and radiotherapy are difficult to avoid tumor resistance and progression. James Allison and Tasuku Honjo discovered the inhibition of negative immune regulation by cancer therapies, for which they were awarded the 2018 Nobel Prize in Medicine (16). This finding enabled tumour immunotherapy to reach unprecedented heights. In recent years, immunotherapy for glioma, including immune checkpoint blockade (ICB), has been considered to be a promising approach (1). However, most patients are not sensitive to ICB treatment (17). Therefore, it is necessary to identify novel biomarkers to combine with immune checkpoints (ICPs) to provide benefits to patient by avoiding immune-related adverse events and decreasing treatment costs. ICD is a new type of regulatory cell death (RCD) defined by the NCCA in 2018 that induces adaptive immunity, thereby enhancing antitumour immunity. Studies have shown that ICD can improve antitumour immune efficacy in combination with immune checkpoints (18). In glioma immunotherapy, identifying discrepancies in response to immune checkpoint blockade across genomic subtypes remains a challenge to overcome (19). Moreover, the use of a single differentially expressed gene as a biomarker is not reliable in individual glioma patients due to the highly heterogeneous character of gliomas (20).

In the present study, we explored the expression, function, and genetic alterations of 34 ICD-related genes. Based on 12 ICD-related genes, we constructed and validated an ICD-related risk signature that serves as a novel prognostic biomarker of LGG patients and might predict ICB immunotherapy response. In addition, the relationship between risk scores and clinicopathological characteristics, immunity profiles, and drug sensitivity was explored separately. Immunohistochemistry verified the expression of several ICD-related genes at the protein level in LGG tissues.

Most differentially expressed ICD-related genes are highly expressed in tumour tissues. Based on consensus clustering, we identified two ICD subtypes through ICD-related gene expression and found that the subtype with high ICD expression was associated with a poor prognosis. Furthermore, we constructed and validated an ICD-related risk signature. Compared with other clinically independent prognostic factors, including grade, age, IDH mutation status, and p/19q codeletion status, the risk signature has a more significant prognostic predictive value, which might effectively stratify patients based on risk and predict individual mortality risk. Of course, a nomogram that combines the risk signature with other clinical factors will have greater predictive value. Given that clinically independent prognostic factors, such as wild type IDH and 1p19q noncodeletion, are usually associated with poor responses to conventional radiotherapy or chemotherapy (17), our results showed that risk scores could distinguish LGG patients based on IDH mutation and 1p/19q codeletion status, indicating that LGG patients with higher risk scores may exhibit an inadequate response to radiotherapy or chemotherapy. Approximately 80-90% of LGG patients harbour IDH1 mutations compared with only 10% in GBM patients (21). IDH1 mutations are associated with a better prognosis in glioma patients. In addition to making tumour cells more sensitive to temozolomide and radiation therapy, IDH1 mutations can also make glioma cells susceptible to DNA damage and apoptosis (21). In our somatic mutation analysis results, there were fewer IDH1 mutations in the high-risk group and ICD-high subtype, which further suggests that traditional treatment may not be effective in patients with high-risk scores. Studies have shown that the presence of CIC mutations is associated with better survival in glioma patients, which is consistent with the better prognosis in our low-risk group of patients (22). Similarly, high-grade glioma has a higher risk score, which suggests that the risk signature can indicate the degree of malignancy and participate in tumor progression.

Given that evading immune destruction is one of the emerging hallmarks of cancer (23), research on the tumour immune microenvironment is increasing. Yoshihara K et al. described an ESTIMATE method to assess the score of stromal and immune cells in tumour samples, and the score was positively related to tumour purity (24). Patients with low-purity LGG tumours typically exhibit an advanced stage and poor prognosis (25). In our study, high-risk patients tended to have higher ESTIMATE scores and lower tumour purity, which could explain why high-risk patients are associated with poor prognosis. T cells follicular helper play an antitumour role in cancers and have potential implications for PD1 and PDL1 immunotherapies (26). NK cells, which serve as the first line of defence against cancer, are strong executors of innate immunity and have the ability to kill circulating tumour cells (27). It has been reported that type M2 macrophages are the main factors that induce an immunosuppressive tumour microenvironment (28). Previous studies have shown that eosinophil cells exert an antitumour cytotoxic response *via* degranulation (29). Mast cells can inhibit or promote several processes in tumour biology that largely depend on the stimulation of the microenvironment (30). Our results revealed that T cells follicular helper, NK cells, and eosinophil cells were significantly downregulated, whereas M2 macrophages were significantly upregulated in the high-risk group. These results indicated that various fractions of immune-inflammatory tumour-infiltrating cells established an immunosuppressive tumour microenvironment in the high-risk groups. Therefore, upregulating the number and activity of infiltrating T cells follicular helper, NK cells, and eosinophil cells and repolarization of M2 into M1 macrophages in tumours represent potentially promising methods to treat high-risk LGG patients.

Furthermore, immune-related functions, including T cell co-stimulation, T cell co-inhibition, checkpoint, HLA, MHC-class-I, were all activated in the high-risk group, indicating that patients in the high-risk group with immune suppression features would respond to immunotherapy (31). Tumours usually evade immune surveillance by downregulating one or more molecules critical for MHC antigen presentation (32). In our GSVA results, the antigen processing and presentation pathway was active in the high-risk group, which further demonstrated the effectiveness of immunotherapy in the high-risk group. Immune checkpoint blockade (ICB) is becoming a main treatment modality given its ability to reverse the signalling of the immunosuppressive Tumour Microenvironment (33). ICP expression is essential for immune escape and ICB treatment, so immune checkpoint inhibitors have gradually become the focus of the latest developments in cancer immunotherapy (34, 35). In the current research, almost all 47 ICPs, including vital ICPs (PD-1, PD-L1, and CTLA4), exhibited significantly higher expression levels in the high-risk groups, suggesting that high-risk patients might exhibit improved sensitivity to ICB therapy. The potential relationship between the ICD-related risk signature developed in this study and immune infiltration and ICPs may represent a promising research direction for improving the efficacy of immunotherapy in solid cancers.

In conclusion, ICD-related genes play an important role in the immune microenvironment. The ICD-related risk signature constructed and validated in this study for predicting LGG patient OS were associated with changes in the LGG tumour immune microenvironment and may predict immunotherapy response. Our study provides a novel and comprehensive perspective to elucidate the underlying mechanisms of LGG prognosis and provides direction for future individualized cancer immunotherapy.

## Data availability statement

The data presented in the study are deposited in the TCGA Database (https://portal.gdc.cancer.gov/repository), accession number: TCGA-LGG; CGGA Database (http://www.cgga.org.cn/download.jsp), accession number: mRNAseq_693 and mRNAseq_325; and GTEx Database (https://xenabrowser.net/datapages/), accession number: GTEX.

## Ethics statement

This study was reviewed and approved by the Department of Neurosurgery, Renmin Hospital of Wuhan University, China (approval number: 2012LKSZ(010)H). Written informed consent was obtained from all participants for their participation in this study.

## Author contributions

JC, QC, and JY designed the research. JC and YH downloaded and analyzed the data. LY, ZY, LG, ST, QS, and YW wrote the paper. All authors read and approved the final manuscript.

## Funding

This work was supported by the National Natural Science Foundation of China (No. 82072764) and Natural Science Foundation of Hubei Province (No. 2020CFB256).

## Conflict of interest

The authors declare that the research was conducted in the absence of any commercial or financial relationships that could be construed as a potential conflict of interest.

## Publisher’s note

All claims expressed in this article are solely those of the authors and do not necessarily represent those of their affiliated organizations, or those of the publisher, the editors and the reviewers. Any product that may be evaluated in this article, or claim that may be made by its manufacturer, is not guaranteed or endorsed by the publisher.

## Supplementary material

Supplementary Figure 1 The somatic mutations in ICD-High and ICD-Low subtypes (obtained by consensus clustering methods). **(A, B)** The top 20 most frequently mutated genes in the ICD-high subtype **(A)** and ICD-low subtype **(B)** were visualized in waterfall plots.

Supplementary Figure 2 The DCA curves of the nomogram, risk, age, gender, grade, and 1p19q codeletion status in predicting 1- **(A)**, 3- **(B)**, and 5-year **(C)** overall survival

Supplementary Figure 3 The relationship between the expression levels of 12 ICD-related risk genes and drug sensitivity in pan-cancer based on data from the CellMiner database.

Supplementary Figure 4 The relationship between the expression levels of 12 ICD-related risk genes and drug sensitivity in pan-cancer based on data from the GSCA database.

Supplementary Table 1 Genes and corresponding coefficients in the ICD-related risk signature.
