# Supplementary figures and images for: Immunogenic cell death-related risk signature predicts prognosis and characterizes the tumour microenvironment in lower-grade glioma
Source: Front Immunol. 2022 Oct 17;13:1011757. doi: 10.3389/fimmu.2022.1011757 (PMC9618960; doi:10.3389/fimmu.2022.1011757)

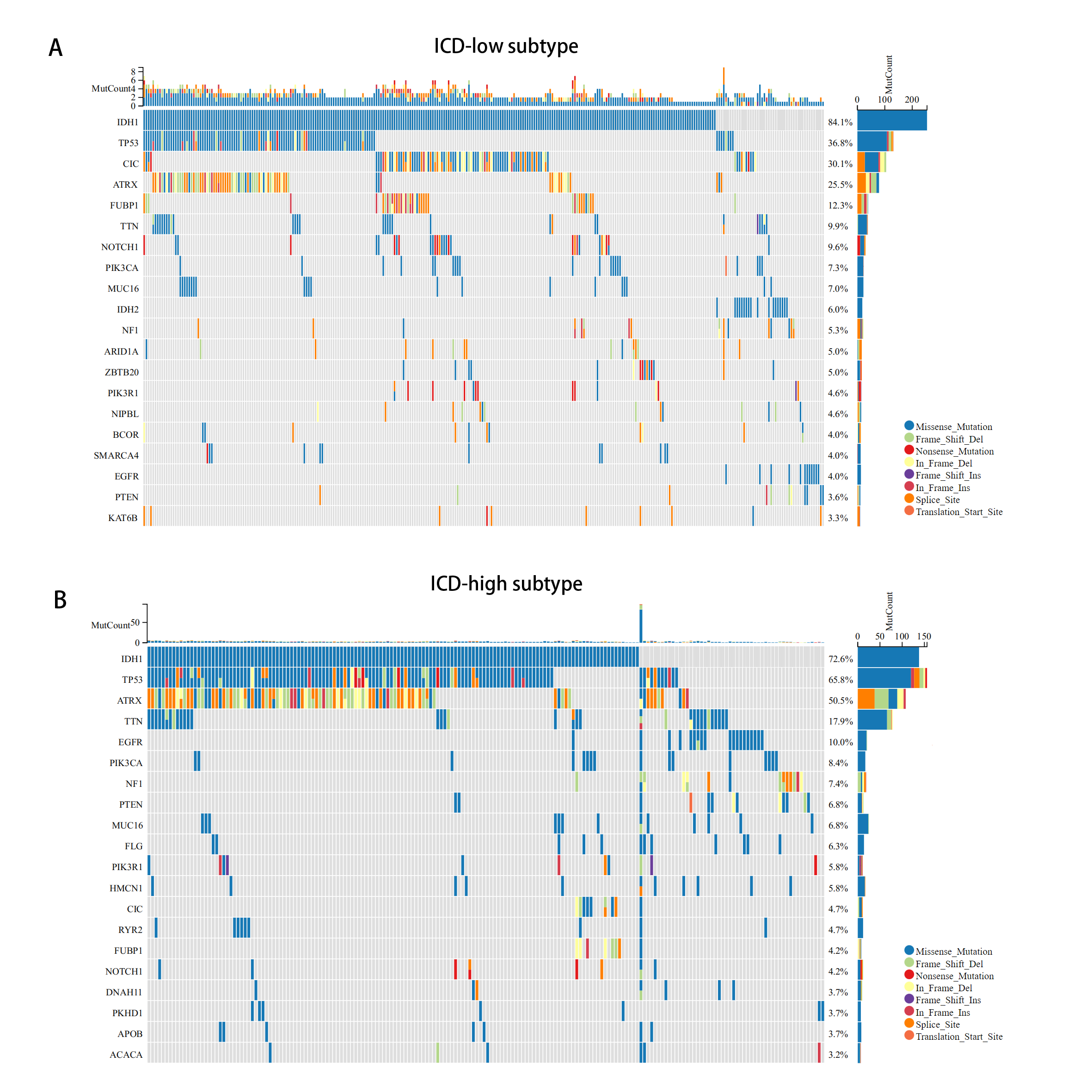

Supplement: Supplementary Figure 1 — The somatic mutations in ICD-High and ICD-Low subtypes (obtained by consensus clustering methods). (A, B) The top 20 most frequently mutated genes in the ICD-high subtype (A) and ICD-low subtype (B) were visualized in waterfall plots. [file Image_1.tif]

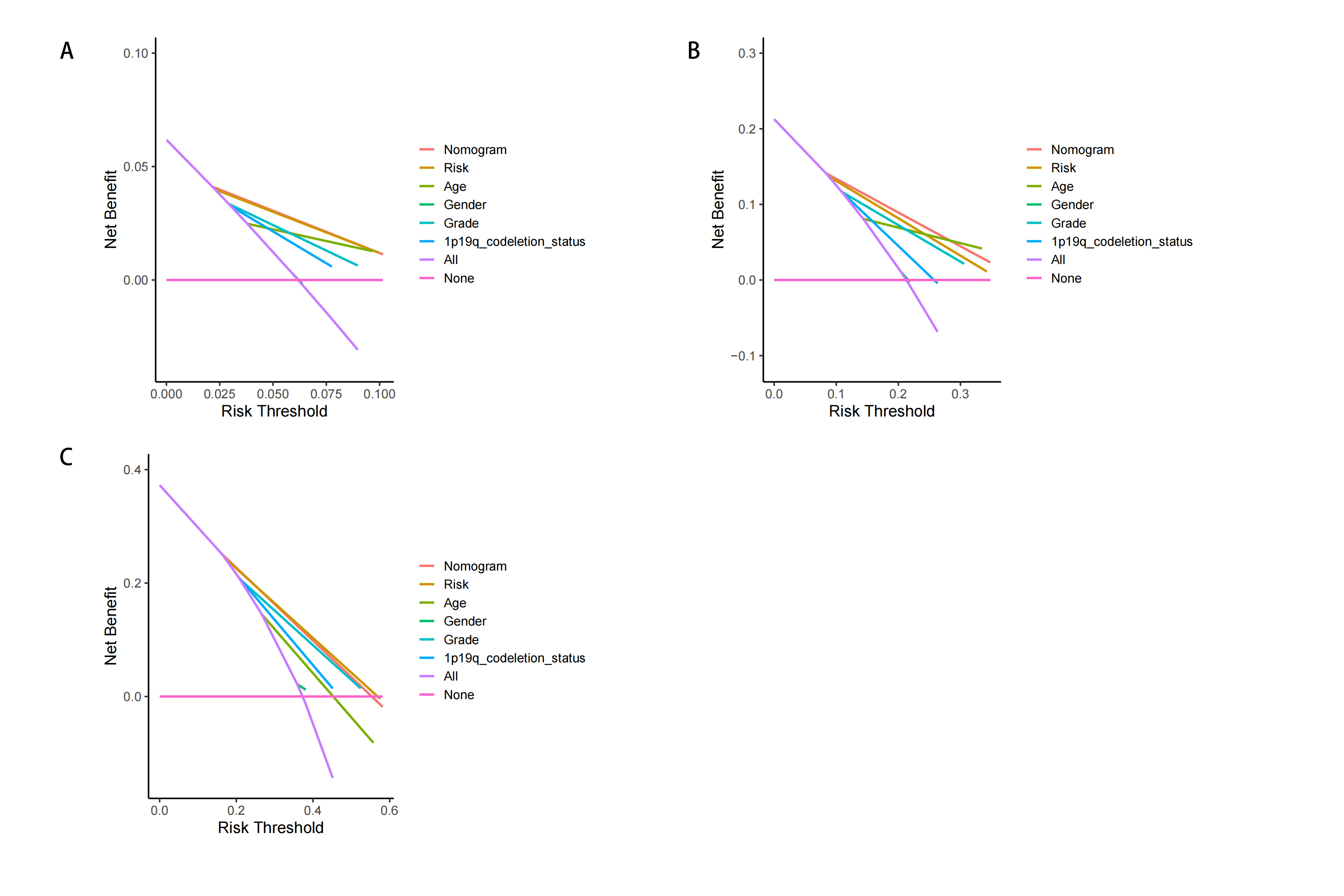

Supplement: Supplementary Figure 2 — The DCA curves of the nomogram, risk, age, gender, grade, and 1p19q codeletion status in predicting 1- (A), 3- (B), and 5-year (C) overall survival [file Image_2.tif]

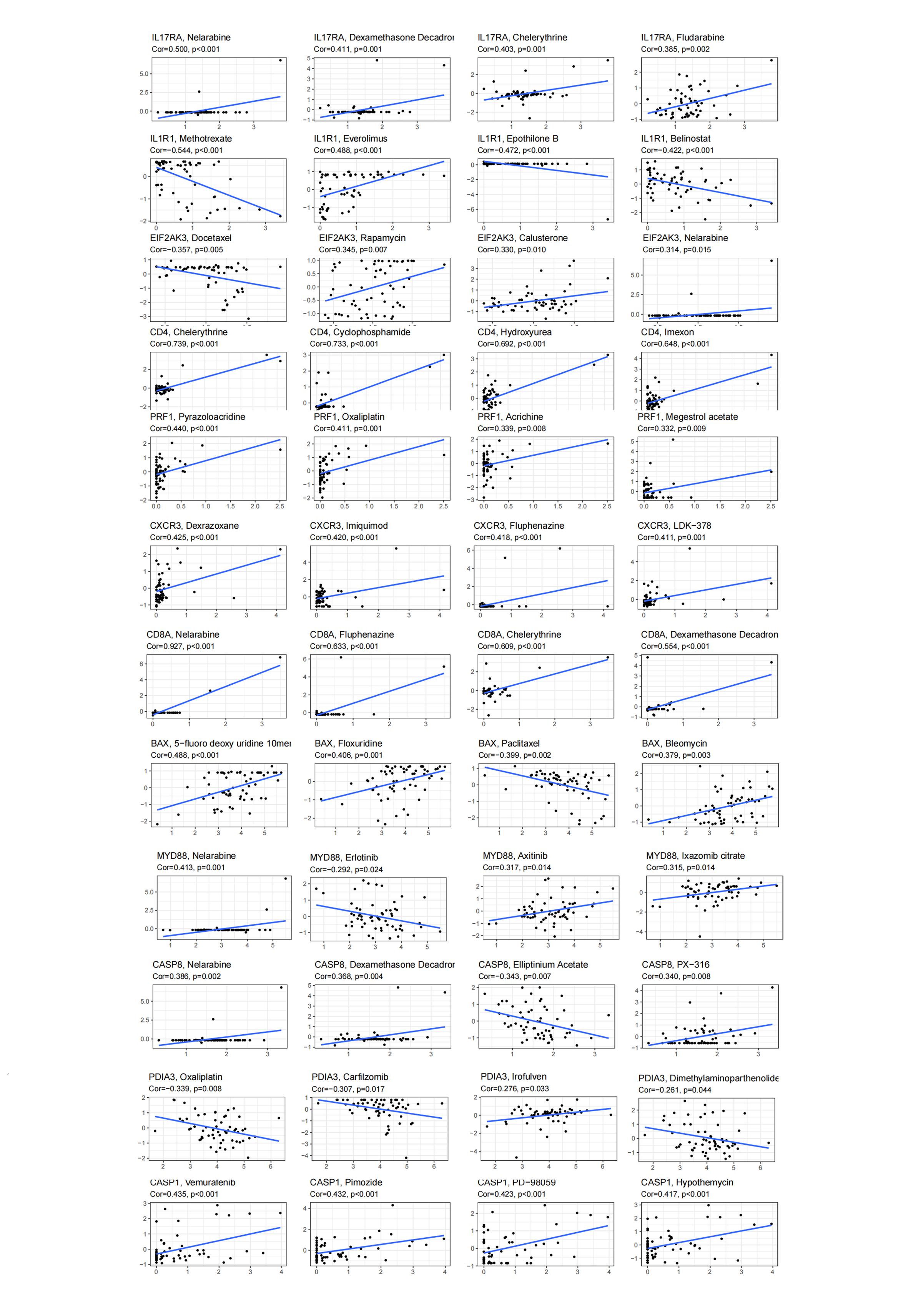

Supplement: Supplementary Figure 3 — The relationship between the expression levels of 12 ICD-related risk genes and drug sensitivity in pan-cancer based on data from the CellMiner database. [file Image_3.tif]

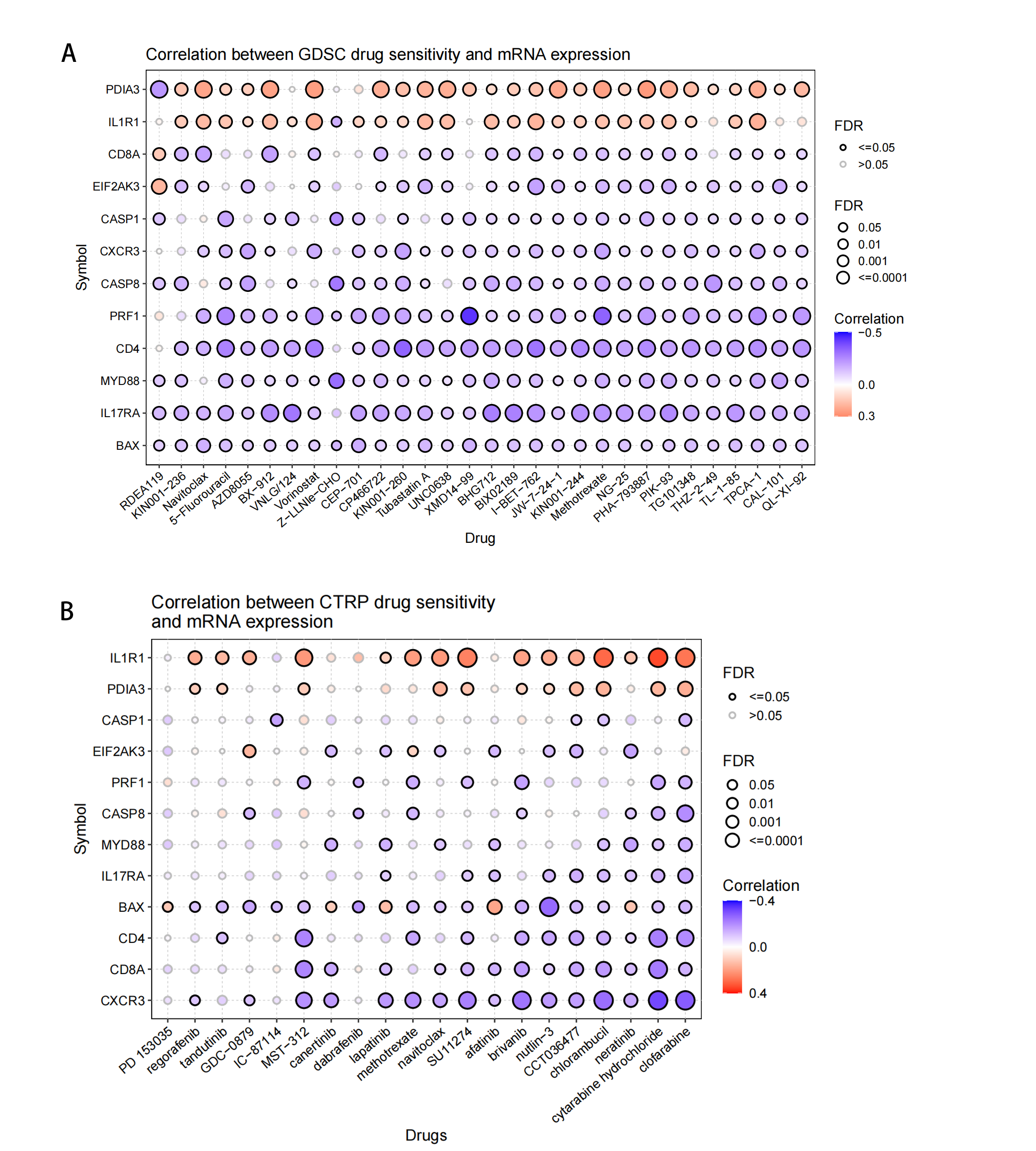

Supplement: Supplementary Figure 4 — The relationship between the expression levels of 12 ICD-related risk genes and drug sensitivity in pan-cancer based on data from the GSCA database. [file Image_4.tif]
