# Supplementary material for: Immunogenic cell death-related risk signature predicts prognosis and characterizes the tumour microenvironment in lower-grade glioma
Source: Front Immunol. 2022 Oct 17;13:1011757. doi: 10.3389/fimmu.2022.1011757 (PMC9618960; doi:10.3389/fimmu.2022.1011757)
Supplement: Supplementary Table 1 — Genes and corresponding coefficients in the ICD-related risk signature. [file Table_1.docx]

Supplementary Table 1. Genes and corresponding coefficients in ICD-related risk signature.

| Gene | Coefficient |
| --- | --- |
| IL17RA | -0.0356475247794726 |
| IL1R1 | -0.130116970276095 |
| EIF2AK3 | 0.57369404874085 |
| CD4 | -0.176213792285495 |
| PRF1 | -0.492704432543956 |
| CXCR3 | -0.361060075046346 |
| CD8A | 0.706414858071808 |
| BAX | 0.206669964847233 |
| PDIA3 | -0.152808017621083 |
| CASP8 | 0.909896849281034 |
| MYD88 | 0.427577645535969 |
| CASP1 | 0.376591469691248 |
